# Progesterone as an Anti-Inflammatory Drug and Immunomodulator: New Aspects in Hormonal Regulation of the Inflammation

**DOI:** 10.3390/biom12091299

**Published:** 2022-09-14

**Authors:** Tatiana A. Fedotcheva, Nadezhda I. Fedotcheva, Nikolai L. Shimanovsky

**Affiliations:** 1 Science Research Laboratory of Molecular Pharmacology, Medical Biological Faculty, Pirogov Russian National Research Medical University, Ministry of Health of the Russian Federation, Ostrovityanova St. 1, Moscow 117997, Russia; 2 Institute of Theoretical and Experimental Biophysics, Russian Academy of Sciences, Institutskaya Str. 3, Pushchino 142290, Russia

**Keywords:** progesterone (P_4_), NF-κB, FKBP51, cyclosporine A (CsA), HSP90, chaperone, tacrolimus

## Abstract

The specific regulation of inflammatory processes by steroid hormones has been actively studied in recent years, especially by progesterone (P_4_) and progestins. The mechanisms of the anti-inflammatory and immunomodulatory P_4_ action are not fully clear. The anti-inflammatory effects of P_4_ can be defined as nonspecific, associated with the inhibition of NF-κB and COX, as well as the inhibition of prostaglandin synthesis, or as specific, associated with the regulation of T-cell activation, the regulation of the production of pro- and anti-inflammatory cytokines, and the phenomenon of immune tolerance. The specific anti-inflammatory effects of P_4_ and its derivatives (progestins) can also include the inhibition of proliferative signaling pathways and the antagonistic action against estrogen receptor beta-mediated signaling as a proinflammatory and mitogenic factor. The anti-inflammatory action of P_4_ is accomplished through the participation of progesterone receptor (PR) chaperones HSP90, as well as immunophilins FKBP51 and FKBP52, which are the validated targets of clinically approved immunosuppressive drugs. The immunomodulatory and anti-inflammatory effects of HSP90 inhibitors, tacrolimus and cyclosporine, are manifested, among other factors, due to their participation in the formation of an active ligand–receptor complex of P_4_ and their interaction with its constituent immunophilins. Pharmacological agents such as HSP90 inhibitors can restore the lost anti-inflammatory effect of glucocorticoids and P_4_ in chronic inflammatory and autoimmune diseases. By regulating the activity of FKBP51 and FKBP52, it is possible to increase or decrease hormonal signaling, as well as restore it during the development of hormone resistance. The combined action of immunophilin suppressors with steroid hormones may be a promising strategy in the treatment of chronic inflammatory and autoimmune diseases, including endometriosis, stress-related disorders, rheumatoid arthritis, and miscarriages. Presumably, the hormone receptor- and immunophilin-targeted drugs may act synergistically, allowing for a lower dose of each.

## 1. Introduction

The goal of this paper is to highlight the possibility of using P_4_ and its derivatives as alternative steroid hormones to glucocorticoids in the treatment of inflammatory diseases, especially chronic inflammatory diseases accompanied by resistance to hormone therapy.

Acute inflammation is simultaneously a typical pathological process and a protective reaction of the body in response to infections and tissue damage. When one of the main regulators of inflammation—the transcription nuclear factor kappa B (NF-κB)—is constitutively activated due to translocation to the nucleus, the inflammation is no longer an acute, rapid protective reaction of the body to pathogens, but in itself is a stimulus for the development of serious pathologies, including cancer and autoimmune diseases [1]. In the case of acute inflammation, the moderate production of proinflammatory cytokines serves as a protective factor, while in chronic inflammation and autoimmune and allergic reactions, it acts as a negative factor for the body’s immune homeostasis. It is well known that steroid hormones are endogenous regulators of inflammation and cytokine production. In particular, glucocorticoids (GCs) are known to be potent immunosuppressive agents with anti-inflammatory activity [2,3]. GCs are the most widely prescribed class of drugs worldwide and their effectiveness in treating acute or chronic inflammation is undeniable [4]. However, there are some limitations to their clinical application. In addition to the gradually emerging resistance to GCs, serious side-effects of their usage are hyperglycemia with the further possibility of insulin resistance, sodium retention, and hypertension, muscle atrophy, osteoporosis, features of Cushing’s syndrome, etc. [5]. The response to GC relies on several factors, including the duration of the stimulus (acute or chronic) and the physiological state of the immune system. In pathological situations, GCs may function as anti-inflammatory molecules to control the process. In contrast, under normal physiological conditions, they may play a proinflammatory role [2,6].

An alternative to GCs can be progesterone (P_4_) due to its anti-inflammatory and immunomodulatory effects. Progesterone at an equal concentration of 0.3–1 ng/mL is present in both men and women. During pregnancy, its concentration increases to 300 ng/mL, and in the luteal phase, it increases to 14 ng/mL. Both in menopausal women and in men over the age of 60, the P_4_ concentration decreases, being equal to 0.38 ± 0.13 ng/mL in men aged 20–90 years and 0.38 ± 0.37 ng/mL in postmenopausal women aged 50–90 years [7]. This fact may explain a more severe course of infectious and autoimmune diseases in people over 60 years old, when the protective effect of P_4_ is reduced.

Recently, some unique anti-inflammatory and immunomodulatory effects of P_4_ have been shown, particularly its anti-inflammatory role in COVID-19 therapy. In 2021, a study was completed on 42 men with severe COVID-19, where the patients were treated with P_4_ in addition to the standard of care. The participants needed 3 days less supplemental oxygen (median, 4.5 vs. 7.5 days) and were hospitalized for 2.5 fewer days (median, 7.0 vs. 9.5 days) as compared with control subjects [8]. A decrease in circulating progesterone levels has been noted in patients with SARS [9]. Sex steroid hormones, particularly P_4_, may be useful in preventing and managing severe COVID-19; related clinical trials are ongoing [10,11]. It was revealed that P_4_ decreases the production of IL-1β, IL-6, TNFα, and IL-12, as well as the production of chemokines (e.g., monocyte chemoattractant protein-1 MCP-1/CCL2); moreover, due to its good tolerability and neuroprotective properties, P_4_ could be a very useful addition to established COVID-19 therapy [12].

The possible side-effects with progesterone usage as an anti-inflammatory drug should be extremely minimal or absent. For progestins with affinity for GR, MR, or AR, some side-effects may be associated with the concomitant glucocorticoid, mineralocorticoid, or androgenic activity, which may be accompanied by water retention, verification, and acne (levonorgestrel, gestodene, drospirenone, and MPA). Progestins dydrogesterone and dienogest have no adverse hormonal activities [13] and could be adequate drugs for the therapy of inflammatory processes.

In general, all progestins are classified as low-toxicity substances, and their usage during pregnancy, as well as in assisted reproductive technologies, testifies to the absence of general and reproductive toxicity [14].

The usage of P_4_ in men is not well studied. P_4_ is the essential hormone for the normal functioning of the male endocrine system, i.e., for spermatogenesis, testosterone biosynthesis in Leydig cells, and neuroprotective function in the brain [15]. For example, the lack of RP expression in germ cells leads to male infertility [16].

The neuroprotective role of P_4_ has been confirmed in both men and women; there are results of a number of clinical studies confirming the high therapeutic efficacy of P_4_ against necrotic damage and behavioral abnormalities caused by traumatic brain injury (TBI) [17,18,19,20].

Importantly, there was no adverse event after the administration of progesterone, at least at 1 mg·kg^−1^ per 12 h, as well as no further late toxicity up to 6 months in both women and men in the trial [18]. Therefore, side-effects should not be expected in men, and progesterone treatment can be a potential therapy not only for TBI, but also for other inflammatory diseases [21], as well as an established pharmacologic therapy for TBI.

Recently, it was shown that intranasal delivery of progestin segesterone acetate may be an efficient strategy to promote recovery after stroke in males and realize cerebroprotection via PR in the brain [22]. Later, the role of mitochondrial metabolism and oxidative protection in segesterone acetate’s therapeutic effects in stroke have been confirmed [23].

Some examples of the anti-inflammatory action of progesterone and progesterone-related drugs on different human and animal models are listed in Table 1.

Many other studies have shown the anti-inflammatory and immunomodulatory effects of P_4_, which are accomplished through P_4_ nuclear and membrane progesterone receptors (PRs) [34,35,36,37,38,39]. The immune tolerance during pregnancy is sufficient to assess its role. P_4_ causes immune tolerance by inhibiting the production of proinflammatory cytokines and reducing the activity of T-helper type 1 cells (Th1) [40]. In contrast, in women with recurrent spontaneous miscarriage, an increase in the level of Th1 cytokines, IL-2, and IFN-γ, and a decrease in the level of Th2 cytokines and IL-10 were noted [41,42]. In this context, it is important to note that P_4_ and its synthetic analogues are successfully used in clinical practice to prevent miscarriage [14]. The receptor-mediated pathways of the anti-inflammatory action of P_4_ are complex, diverse, and not well studied. The role of chaperones and PR coactivators, as well as new therapeutic strategies for their regulation, has not been previously elucidated, although there is strong evidence for their involvement in the anti-inflammatory and immunomodulatory effects of P_4_.

In this review, we consider the main mechanisms of the anti-inflammatory and immunomodulatory effects of P_4_, and we suggest new strategies for the enhancement of these effects when needed, e.g., when GC resistance or P_4_ resistance occurs. Essentially, P_4_ acts on both cellular and humoral immunity since it regulates T-cell activation and the production of anti-inflammatory cytokines, as well as inhibits the expression of immunophilins and heat-shock proteins, factors that contribute to the development of inflammation. The current analysis of the anti-inflammatory effects of P_4_, with a special attention to the role of the progesterone receptor (PR) chaperones—FKBP51, FKBP52, and HSP90—in the implementation of P_4_ is presented in this paper. The exact role of FKBP51 and FKBP52 in hormone signaling and hormonal resistance is actively being explored, as well as the regulation of their mRNA expression and post-translational changes [43]. The main mechanisms of P_4_ anti-inflammatory and immunomodulatory action, as well as the possible strategies for enhancing GC or P_4_ anti-inflammatory activity using FKBP-targeted drugs, such as tacrolimus or cyclosporine A, are discussed.

## 2. Progesterone Regulates T-Cell Activation and Cytokine Production by Immune Cells

The progesterone-specific regulation of T-cell activation became evident through several observed facts: a more active cell-mediated immune response to viral infections in women than in men; the correlation of peak incidence of autoimmune pathology in women with periods of hormonal changes, i.e., puberty, early postpartum, or menopause; the phenomenon of immune tolerance during pregnancy, when the concentration of P_4_ increases hundredfold [44]. It is believed that T-cell activation can be specifically inhibited by P_4_, but not by estrogens. Such a conclusion can be made as, in 1990, it was shown for the first time that the in vitro cytotoxic T lymphocyte (CTL) response was strongly inhibited by P_4_ but not by estrone, estradiol, or testosterone [45]. Recently, this observation was confirmed [46]. It should be noted that, in both studies, P_4_ inhibited T-cell activation at high concentrations on the order of µM.

This specific P_4_-dependent regulation may be due to the P_4_-dependent production of at least two mediators of the immune response: PIBF (progesterone-induced blocking factor) and LIF (leukemia inhibitory factor) [26]. *PIBF* is one of the progesterone-regulated genes [47,48,49]. PIBF is secreted by cells expressing PR, i.e., immune cells and host cells, while cancer cells also produce PIBF, e.g., MCF-7 [50]. Progestins bind to PRs located in immune cells, including NK, macrophages, dendritic cells, and T cells, as well as in non-immune cells, such as epithelial and endothelial cells. Then, PIBF is secreted, binds to the PIBF receptor, and activates signaling pathways that lead to the appropriate cytokine production [51]. P_4_ induces PIBF in leukemia cell lines, and progesterone receptor antagonist mifepristone decrease PIBF mRNA and protein synthesis [48].

It was shown that PIBF is overexpressed by tumor cells and by highly proliferating normal cells; PIBF is also copurified with centrosomes. PIBF can be a promising marker of pregnancy pathology (if not found in urine) or malignant processes (if a great amount of PIBF is found in urine) [50,52].

Urine concentrations of PIBF and serum concentrations of anti-inflammatory cytokines were lower in women with threatened spontaneous abortion than in healthy women, while proinflammatory (IL-6, TNF-α, IFN-γ) cytokines were higher in the group with lower PIBF [53].

In general, PIBF modulates cytokine synthesis and T-cell cytotoxicity toward a more immunotolerant state. CD4^+^ and CD8^+^ T cells have been found to respond to progesterone in a dose-dependent manner, causing marked changes in T-cell cytokine secretion [54].

LIF is a member of the IL-6 cytokine family and is expressed in almost every tissue type within the body [55]. LIF has its own receptors; it regulates implantation, as well as the repair processes of nerve, bone, and muscle. The LIF action is tissue-dependent, but a common feature is the stimulation of differentiation [56].

LIF is a cytokine that is induced by estrogen and P_4_ [57]. LIF is produced under the action of IL-4, whose level increases under the action of P_4_. LIF is an anti-inflammatory and analgesic cytokine since it reduces interleukin IL-1β and nerve growth factor (NGF) expression. Conversely, a local injection of low doses of recombinant LIF diminishes the mechanical and thermal hypersensitivity of the skin [58].

**Figure 1:**
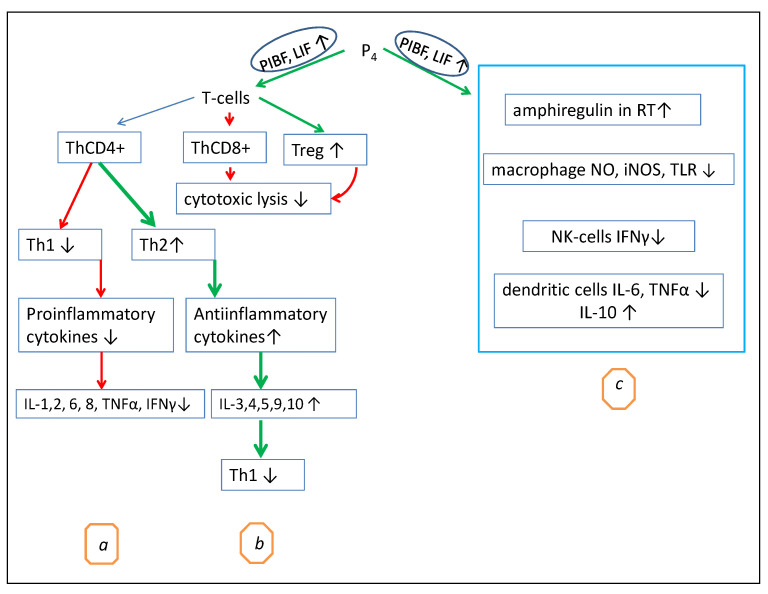
The proposed mechanisms of the P_4_ immunomodulatory effects. (**a**) P_4_ induces PIBF and LIF production, which inhibit the Th1 pathway and reduce the production of proinflammatory cytokines; (**b**) P_4_ upregulates Th2 differentiation and anti-inflammatory cytokine synthesis, which downregulate Th1 differentiation; P_4_ decreases CD8^+^ T cytotoxicity and upregulates Tregs, which also decrease CD8^+^ T cytotoxicity; (**c**) by stimulation of the secreting amphiregulin, P_4_ activates proliferation and repair in the respiratory tract (RT). P_4_ decreases the production of proinflammatory cytokines IFN-γ, IL-6, and TNF-α by NK (natural killer) and dendritic cells, as well as decreases NO synthesis by macrophages. Red arrow—inhibition, green arrow—stimulation.

PIBF and LIF play a direct role in T-lymphocyte differentiation (Figure 1). Both cytokines inhibit the Th1 pathway, reduce the production of proinflammatory cytokines, modulate the T-cell receptor signaling and cellular cytotoxicity managed by T killer cells, and enhance the differentiation of regulatory T cells (Tregs), thus promoting immune tolerance [59].

Cytokines PIBF and LIF, especially in the uterus, placenta, and bones, and epidermal growth factor amphiregulin (AREG) in the lungs [60] are the main mediators of the regulatory role of P_4_ in T-cell activation. The proposed mechanisms of the P_4_ immunomodulatory effects are summarized in Figure 1.

As shown in Figure 1, possibly through PIBF and LIF, P_4_ decreases Th1 differentiation and CD8^+^ cytotoxicity and stimulates CD4^+^ T helper differentiation in a manner to decrease the amount of Th1 and increase the amount of Th2 [61]. As a consequence, the Th1-mediated production of proinflammatory cytokines decreases, while the Th2-mediated production of anti-inflammatory cytokines increases (Figure 1). High concentrations of P_4_ can stimulate the production of anti-inflammatory cytokines, particularly IL-4 and IL-10, by CD4^+^ T-helper cells, leading to Th2-type anti-inflammatory responses [12].

P_4_ can influence T-cell activation through PIBF and LIF secretion, regulating it via membrane PR (mPR). It is known that T-cell activation is associated with a rapid increase in the intracellular free calcium concentration. This process is thought to be associated with mPR, and P_4_ is capable of regulating the intracellular free calcium concentration [62,63]. The activation of PGRMC1 (progesterone receptor membrane component 1) by P_4_ attenuates this Ca^2+^ signaling pathway, which leads to a decrease in the Ca^2+^-dependent NFAT1 nuclear accumulation [63]. It is probable that this P_4_ and PRGMC1-related pathway can suppress T-cell activation.

Furthermore, the inhibition of T-cell activation by P_4_ can be indirect. P_4_ enhances the induction of IL-10-producing cells, particularly dendritic cells [19]. Thus, IL-10 may inhibit Th1 differentiation [64]. Figure 1 illustrates the main immunomodulatory effects of P_4_. This specific immunosuppressive action of P_4_ is associated with the inhibition of the Th1 and Th17 pathways, which can shorten the active course of some chronical autoimmune diseases, such as rheumatoid arthritis and multiple sclerosis [65]. Hormone replacement therapy (HRT) can also be an example of the anti-inflammatory action of P_4_ since this hormone reverses the menopausal CD4/CD8 ratio, as well as limits the general peri- and postmenopausal inflammatory state [66]. 

In addition to its own inhibition of T-cell activation, P_4_ has a significant effect on dendritic cells, NK cells, and macrophages (Figure 1C). In macrophages, P_4_ decreases the expression of iNOS, NO, and TNFα and the release of prothrombotic factors [65]. In dendritic cells, P_4_ decreases CD40, CD80, CD86, TNF-α, IL-1β, IL-6, and IL-12 and increases IL-10, IL-8, and CD11c [39]. It is also shown that at low doses, P_4_ increases IFN-α, while at high doses, P_4_ and progestin MPA decrease IFN-α [39]. In NK cells, P_4_ reduces their cytotoxicity and the production of IFN-γ, as well as stimulates caspase-dependent apoptosis [65]. NK cells expressing the PR are especially susceptible to progesterone-induced apoptosis. High levels of P_4_ produced by the human placenta are likely to induce two factors at the maternal–fetal interface that inhibit NK cytolytic activity and IFN-γ secretion, namely, major histocompatibility complex class IG (HLA-G) and the above-mentioned PIBF. It was shown that P_4_ can directly induce apoptosis and suppress IFN-γ production in human peripheral blood NK cells in vitro, possibly via intracellular PRs [65].

The exact mechanism via which P_4_ regulates T-cell activation is still unclear. Recent studies using the analysis of P_4_-induced transcriptomic changes in an in vitro model of human CD4^+^ T cells demonstrated a profound direct effect of P_4_ on T-cell activation and downregulation of the immune genes involved in autoimmune diseases such as multiple sclerosis and rheumatoid arthritis [67].

PRs are widely distributed throughout the body; the effects of natural and synthetic forms of P_4_ are not limited to the reproductive tract, but can occur at other mucosal sites, including the respiratory and gastrointestinal tracts [39]. PRs are expressed in the uterus, ovary, lung, brain, testis, prostate and mammary gland, and immune cells. PRs are located on most immune cells (neutrophils, eosinophils, basophils, mast cells, monocytes, macrophages, dendritic cells, NK cells, and lymphocytes (B cells and T cells)) [68]. Thus, the P_4_ effects on immune suppression are realized by binding to the PRs on immune cells.

The anti-inflammatory and immunomodulatory action of P_4_ is associated with the inhibition of the proinflammatory cytokines [69]. P_4_ mostly reduces the amount of proinflammatory cytokines and induces a certain amount of anti-inflammatory cytokines. Table 2 lists the cytokines regulated by P_4_.

In both in vivo and in vitro models, P_4_ has been shown to induce higher levels of anti-inflammatory cytokines such as IL-10 and IL-4, while reducing the production of proinflammatory cytokines such as IL-6, TNF-α, and IL-1β [39,79,80,81]. The effect of P_4_ on the production of these cytokines has also been confirmed during HRT with synthetic progestins in menopausal women [66].

However, it should be noted that the production of proinflammatory cytokines during acute inflammation as a protective reaction of the body can be considered a positive effect on the immune system. That is why cytokines such as IL-6 or IFN-γ can be considered to be both proinflammatory and anti-inflammatory, and the action of antiviral drugs can enhance their production [82]. For example, proinflammatory IFN-γ may have anti-inflammatory effects by inhibiting the production of proinflammatory IL-1 and IL-8, enhancing the production of cytokine antagonists such as IL-1 receptor antagonist (IL-1ra) and IL-18-binding protein (IL18BP), inducing the expression of members of the SOCS regulatory protein family, and activating the apoptosis of leukocytes. This is especially evident in chronic inflammation, such as rheumatoid arthritis.

IFN-γ is constitutively produced as an innate immune response, predominantly by natural killer (NK) and natural killer T (NKT) cells. In the case of an antigen-stimulated immune response, IFN-γ is produced by Th1 CD4 and CD8 cytotoxic T lymphocyte effector T cells. Thus, in this context, IFN-γ can be considered as an anti-inflammatory cytokine [83]. IFN-γ has antiviral, immunoregulatory, and antitumor properties, and its pro-inflammatory effects are manifested as an acute adaptive response of the organism [84].

Nevertheless, P_4_ can have both stimulatory and inhibitory effects on IFN-γ production. In a clinical study that included patients with miscarriage and thin endometrial syndrome, the intracellular production of IFN-γ by endometrial lymphocytes sharply decreased eightfold compared with the group of healthy persons. After treatment with intravaginal micronized progesterone (Luteina), an increase in endometrial thickness was observed (M-echo from 5.9 ± 0.1 to 10.2 ± 0.2 mm), and the level of IFN-γ CD56^+^ induced by endometrial lymphocytes significantly increased [85].

On the contrary, a meta-analysis of the results (I^2^ = 86.2%, *p* = 0.000) of seven studies reporting the expression level of IFN-γ after treatment with the synthetic progesterone analogue PR agonist dydrogesterone showed that dydrogesterone lowered the level of IFN-γ, much more effectively than P_4_ [79]. It was shown on models of human lymphatic endothelial cells (LECs) and mouse lymph nodes that P_4_ and its metabolites reduced TNF-α and IFN-γ production in CD4^+^ and CD8^+^ T cells [86]. A decrease in the synthesis of IFN-γ by NK cells under the influence of P_4_ was also noted in human peripheral blood mononuclear cells (PBMCs) obtained from healthy donors [87]. Thus, P_4_ reduces the production of IFN-γ, and this may be due to the action of PIBF, since anti-PIBF-treated mice have a significantly higher percentage of spleen cells that express IFN-γ [88].

As for the action of P_4_ on the multifactorial TGFβ, the signaling of which plays a role in cell proliferation, differentiation, and apoptosis, there are also no unequivocal data. TGFβ may act as a proinflammatory cytokine in chronic inflammation, as well as promote proliferation and repair in wound healing. TGFβ is potentially an immune modulator, and its signaling is upregulated in many pathological states, especially cancer and fibrosis. In fibrosis, TGFβ blockade suppresses epithelial–mesenchymal transition, inhibits fibroblast differentiation and proliferation, and prevents excessive elaboration of the extracellular matrix [89]. In turn, P_4_ inhibits TGFβ production [90]. Accordingly, P_4_ and progestins decrease the expression of TGFβ target genes SMAD, P38, NFKBP, RAS, and PI3K [91]. The inhibition of TGFβ production by high-dose P_4_ is associated with a decrease in phosphatidylinositol 3-kinase/protein kinase B (PI3K/AKT) signaling since this pathway is activated by TGFβ. There is evidence indicating that the inhibition of the TGFβ signaling pathway may be beneficial in autoimmune disorders, such as multiple sclerosis, through the downregulation of the TH17 pathway [89,91].

As high doses of progestins inhibit TGFβ expression in cancer cells, this also introduces an additional benefit of the use of progestins as anticancer and anti-inflammatory drugs. A negative regulation of IL-6 and IL-1 production by progestins, which has been confirmed in many studies, as mentioned above, suggests the possibility of their usage in anti-COVID-19 therapy, since the monoclonal antibodies targeting these interleukins (anakinra and tocilizumab, respectively) represent the first-stage target therapy of COVID-19 [92].

## 3. P_4_ Inhibits NF-κB Activation

The nuclear factor kappa B (NF-κB) family of transcription factors controls the expression of important regulatory genes responsible for immunity, inflammation, death, and cell proliferation. The NF-κB protein is located in the cytoplasm and can be activated by various cellular stimuli.

These cellular stimuli induce the interaction of NF-κB protein with the IκB kinase (IKK) complex, which then leads to the phosphorylation of IκB, followed by ubiquitination and degradation. After IκB phosphorylation and degradation, the remaining NF-κB p65/p50 dimer is translocated to the nucleus, where it binds to the DNA consensus sequence of various target genes [93,94].

P_4_ inhibits the NF-κB pathway in a different manner: directly, via physical binding of the RelA (p65) subunit; and indirectly, through signal proteins TGFβ and immunophilins FKBP51 and FKBP52. As shown on LPS-stimulated BV-2 microglia cells, P_4_ pretreatment attenuated LPS-stimulated *TNF-α*, *iNOS*, and *COX-2* expression in a dose-dependent fashion [95]. P_4_ suppressed the LPS-induced NF-κB activation by decreasing phosphorylation of the inhibitory κBα (IκBα), decreasing NF-κB p65 subunit phosphorylation, and inhibiting p65 nuclear translocation. P_4_ also decreased the LPS-mediated phosphorylation of p38, c-Jun N-terminal kinase, and extracellular regulated kinase. These specific effects of P_4_ were inhibited by its antagonist mifepristone [95].

It was shown that the p65 subunit specifically inhibits the hormone-induced transactivation of PR [96]. The inhibition is mutual, since PRs were shown to suppress the transcriptional activity of p65. This is due to their direct physical interaction. An in vitro examination showed that the two proteins have the capacity for direct physical interaction. Such a complex could either (i) be unable to bind DNA or (ii) result in the formation of inactive complexes preventing the interaction with essential co-factors or the basal transcription machinery [96]. Then, this trans-repression between p65 and PR could play an important role in immunosuppression, tumorigenesis, and inflammation.

PR is able to increase NFKBPia (a gene encoding NF-κB inhibitor α (NFκBIA)) expression by directly binding to its promotor in uterine myometrial cells [97]. When NF-κB is bound by NFκBIA, it loses its transcriptional activity.

Later it was shown that P_4_ treatment impaired in vivo binding of p65 to both proximal and distal promoters, and P_4_ binding with PR antagonized cytokine-induced *COX-2* gene transcription [98]. P_4_ diminished the IL-1β-stimulated recruitment of NF-κB p65 to both proximal and distal NF-κB elements of the *COX-2* promoter. Thus, the progesterone-mediated decrease in p65 binding to the *COX-2* promoter might be caused by a direct physical interaction of PR with p65. It was found that not only P_4_, but also PR can cause the ligand-dependent and ligand-independent suppression of *NF-κB*. In human fundal myometrial cells, as well as in human breast cancer (T47D) cells, IL-1β alone caused a marked upregulation of *COX-2* mRNA, whereas treatment with P_4_ suppressed this induction [98]. In both cell lines, this inhibitory effect of P_4_ was blocked by PR antagonist mifepristone.

It was revealed using small interfering RNA-mediated ablation of both PR-A and PR-B isoforms that, in the absence of external P_4_, *NF-κB* activation and *COX-2* expression in T47D cells were greatly enhanced. Thus, PR may inhibit *NF-κB* activation of *COX-2* gene expression and uterine contractility via ligand-dependent and ligand-independent mechanisms [98].

As the genes of *TNF-α, iNOS, Cox-2,* and *PTGS2* are known transcription targets of NF-κB, these target genes could be also inhibited by P_4_ and progestins. Similarly, the downregulation of *PTGS2* is important during ovulation, when the basal temperature is rapidly increased; thus, it can induce inflammatory processes. Due to the specific inhibition of Ptgs2, P_4_ has an important protective function, preventing inflammation during ovulation. PR when bound with P_4_ downregulates *PTGS2* expression in granulosa cells. By inhibiting NF-κB, PR reduces *PTGS2* expression and PGE2 synthesis in gonadotropin-induced granulosa cells during ovulation. The lack of PR expression in ovarian granulosa cells has been shown to lead to a hyperinflammatory state characterized by excessive PGE2 synthesis, immune cell infiltration, oxidative damage, and neoplastic transformation of ovarian cells [99].

## 4. Progesterone Regulates Inflammation via FKBP51

The action of P_4_ is realized through nuclear, mitochondrial, and membrane progesterone receptors. The active cytosolic/nuclear progesterone receptor complex exists in dynamic interactions with chaperones HSP90, HSP70, and p23, as well as cochaperone immunophilins FKBP51 and FKBP52 [100]. Studies have implicated alterations in the expression or function of PR coactivators, chaperones, and cochaperones that are bound to the mature form of the cytosolic PR before activation and nuclear translocation in the development of P_4_ resistance [101].

P23 is a small acidic protein with intrinsic molecular chaperone activity. It is best known as a cochaperone of the major cytosolic molecular chaperone Hsp90. P23 inhibits both the basal and the substrate-stimulated ATPase activity of Hsp90, resulting in its stabilization [102]. Hence, targeting p23, as well as other members of the active PR complexes, can modulate PR- and GR-mediated responses. p23 functioning is not as well studied as FKBP51 and FKBP52 functioning.

**Figure 2:**
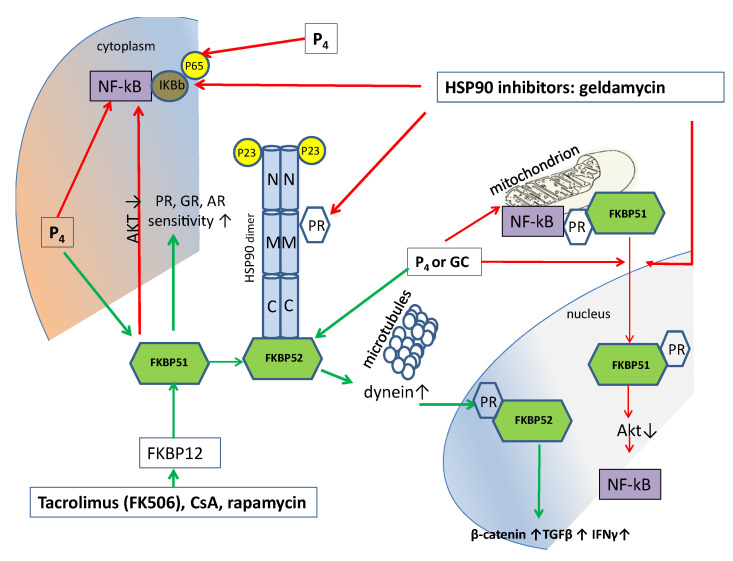
The probable mechanism of the P_4_-mediated PR and GR sensitization by FKBP-targeted drugs. Tacrolimus (FK506), CsA, and rapamycin bind FKBP12 and then inhibit FKBP51, which inhibits supplantation of FKBP52; in this way, FKBP52 does not induce dynein and disturbs FKBP52-mediated signaling (β-catenin, TGFβ, and IFN-γ). Simultaneously, FKBP51 inhibits NF-κB through an Akt-inhibitory mechanism. P_4_ induces FKBP51, thus activating silenced PR, GR, and AR signaling. HSP90 inhibitors reduce the activation of FKBP52-mediated signaling. HSP90 inhibitors also decrease FKBP51 shuttling from the mitochondria to the nucleus; thus, NF-κB is not activated in the nucleus. Red arrow—inhibition, green arrow—stimulation.

FKBP51 and FKBP52 are members of the immunophilin protein family, named because they bind the immunosuppressive drugs tacrolimus FK506, sirolimus (rapamycin), and cyclosporin A (CsA), which play a role in immune regulation and basic cellular processes involving protein folding and trafficking. FKBP51 has *cis*–*trans* prolyl isomerase activity, which is inhibited after FKBP51 binding using the abovementioned immunosuppressive drugs. Binding with immunosuppressants also mediates the calcineurin inhibition. FKBP51 interacts with mature progesterone receptor hetero complexes, as well as glucocorticoid and mineralocorticoid complexes with HSP90 and p23 proteins (Figure 2). Via a short feedback loop, FKBP51 regulates hypothalamic–pituitary–adrenal axis activity. FKBP51 expression is increased by GC or P_4_ and acts on GR or PR in an inhibitory manner; then, cortisol synthesis is decreased, which downregulates inflammation [103].

FKBP51 has the potential to control inflammation in steroid-insensitive patients in a steroid-dependent and -independent manner; thus, it may be worthy of further study as a drug target. Its role in inflammation has been extensively studied, especially in the regulation of the IKK complex, NF-κB signaling, etc., with a focus on its role in hormone-resistant inflammation. It is assumed that the downregulation of FKBP51 expression can restore steroid sensitivity.

For example, the blockade of FKBP51 in a bronchial epithelial cell line resulted in a tenfold increase in the effectiveness of dexamethasone in suppressing IL-6 and IL-8, while the overexpression of FKBP51, on the contrary, reduced sensitivity to prednisolone in a model of pneumonia in mice [104]. FKBP51 silencing also decreased NF-κB translocation to the nucleus [104].

It can be hypothesized that FKBP51 inhibition would increase hormone sensitivity and attenuate inflammation. FKBP51, unlike FKBP52, does not bind to dynein and, thus, does not move GR into the nucleus [103]. That is, FKBP51 overexpression inhibits GR signaling. Upon steroid binding, the steroid receptor heterocomplex exchanges FKBP51 for FKBP52. After this, FKBP52 is able to interact with dynein and further promotes the transcriptional activity of nuclear steroid receptors [105]. It is assumed that, by regulating FKBP51 expression and post-translational changes, it is possible to increase or decrease the response to P_4_ or GC during inflammatory processes [106].

As FKBP51 was described as a regulator of NF-κB (nuclear factor binding near the κ light-chain in B cells) signaling in different cell types, it has been suggested as a drug target for the treatment of NF-κB-mediated inflammation and cancer [107]. FKBP51 interacts with the subunits of the IKK complex [107]. Therefore, FKBP51 ligands can modulate the PR-mediated influence on inflammation, as well as restore the sensitivity to steroid hormones.

FKBP51 ligands can reduce inflammation and proliferation. It was shown that the FKBP51 ligand FK506 inhibits both PR and GR found in T47D breast cancer cells [108]. When needed, this feature can be used to protect against the action of GR, PR, or possibly AR. Indeed, in LNCaP (prostate cancer) cells, another FKBP51 ligand CsA was a highly effective inhibitor of both AR-dependent and -independent proliferation, while FK506 inhibited only AR-dependent growth. Indeed, some studies have shown that AR can be a molecular target for both CsA and FK506 [108].

The localization and functioning of FKBP51 are still under investigation. It is assumed that FKBP51 is a mitochondrial protein present in various cell lines and rat organs, which undergoes nuclear–mitochondrial shuttling [109]. It was also shown that FKBP51 forms complexes in the mitochondria with the glucocorticoid/progesterone receptor and with the Hsp90/Hsp70-based chaperone heterocomplex [109]. Furthermore, FKBP51 is an inhibitory cochaperone for PR, AR, and GR. Conversely, FKBP52 is a stimulatory cochaperone for these receptors. The cytoplasmic FKBP52 is localized to microtubules and, after hormone binding, activates dynein, which moves the mature GR or PR complex to the nucleus for downstream genomic signaling [103] (Figure 2). Thus, FKBP51 is responsible for the binding to the ligand, while FKBP52 is responsible for the transfer of the receptor–ligand complex to the nucleus via dynein binding [105]. Hence, immunosuppressants such as tacrolimus revealed potential therapeutic benefits for their use in treating female infertility due to the enhanced progesterone receptor sensitivity [103].

The role of FKBP51 in the regulation of glucocorticoid-mediated inflammation is also the subject of interest. FKBP51 may provide an additional instrument to regulate the glucocorticoid-mediated pathways especially in the case of pathologies associated with high glucocorticoid levels, where the suppression of the FKBP51 function may help to restore the anti-inflammatory action of glucocorticoids [6]. Therefore, FKBP51 functioning is still not fully studied, but FKBP51 targeting may be promising in inflammation, cancerogenesis, and stress-related disorders. P_4_ and its analogues should be strong regulators of FKBP51 activity, but the exact mechanisms of regulation remain to be explored. FKBP51 ligands such as tacrolimus or CsA can cause immunosuppressant and anti-inflammatory action not only via calcineurin inhibition, but also by increasing sensitivity to steroid hormones. P_4_ and synthetic progestins bind FKBP51 and regulate its expression and activity.

FKBP51 also acts as a negative regulator of AKT since FKBP51 is responsible for binding indirectly through Hsp90 to AKT [110]. Consequently, we can assume that, upon FKBP51 binding with P_4_ or immunodepressants, Akt signaling is decreased, which can be an additional mechanism to increase progestin or GK sensitivity.

However, there are conflicting data on this issue. It is possible that the effect of progestins on FKBP51 is different depending on the dose of progestin and the time of exposure. For example, an inducing effect of the PR agonist R5020 and P_4_ itself on FKBP51 mRNA expression, leading to resistance to P_4_, has been demonstrated [111]. The FK506-binding immunophilin FKBP51 is transcriptionally regulated by progestin and attenuates progestin responsiveness [112].

The opposite action of FKBP51 on the responsiveness to P_4_ was also demonstrated [113]; FKBP51 decreased cell proliferation and increased progestin sensitivity of human endometrial adenocarcinomas by inhibiting Akt. Furthermore, FKBP51 overexpression in progesterone receptor-positive Ishikawa cells sensitized them to medroxyprogesterone acetate (MPA; progestin) treatment by repressing Akt signaling.

As observed, FKBP51 levels are lower in endometrial adenocarcinoma than in normal endometrium tissues [113]. Conversely, FKBP51 expression is higher in the secretory-phase endometrium than in the proliferative phase. Furthermore, FKBP51 expression is higher in younger women than in older women. Thus, the interaction between P_4_ and FKBP51 and the influence of P_4_ on FKBP51 expression and functioning should be more extensively studied with respect to their role in acute and chronic inflammation, as well as in hormone-resistant inflammation and cancer.

## 5. Progesterone Inhibits HSP90, an ATP-Dependent Chaperone

As mentioned above, the active PR complex includes HSP90 protein. HSP90 belongs to the group of high-molecular-weight HSPs, which are ATP-dependent chaperones that require cochaperones to modulate their conformation and ATP binding. Some of these are expressed constitutively, whereas the expression of others is induced by stressful conditions. Even in the absence of stress, HSPs play key roles in living systems by acting as chaperones. They assist in (i) the folding of newly synthesized polypeptides, (ii) the assembly of multiprotein complexes, and (iii) the transport of proteins across cellular membranes [114].

Hsp90 is a central regulator of cellular proteostasis, involved in the maturation of a plethora of substrates (“clients”), including protein kinases, transcription factors, and E3 ubiquitin ligases. Hsp90 is one of the most abundant proteins in the cell [102]. GR and PR, as well as kinases such as v-Src, are the most intensively studied Hsp90 clients [115].

HSP90 is a stabilizing chaperone for many kinases involved in cancer cell signaling, JAK2 signaling, and NFKBP signaling [116]. HSP90 also interacts with and stabilizes the receptor-interacting kinase (RIP). Upon stimulation by TNF-*α*, RIP is recruited to the TNF receptor, thereby allowing the activation of NF-*κ*B, a key component of the inflammatory response. Following depletion of HSP90, RIP is degraded, and the activation of NF-*κ*B is prevented [117]. In this context, the inhibition of the HSP90-dependent pathways in cancers and inflammatory diseases is a promising strategy.

Currently, there are few classes of HSP90 inhibitors; historically, the first of these were geldanamycin and radicicol. Geldanamycin is an ansamycin derivative benzoquinone, which binds to the ATP-binding “pocket” of HSP90. Immunosuppressants 7-allylamino-17-demethoxygeldanamycin (tanespimycin, 17-AAG) and 17-[2-(dimethylamino) ethyl] amino-17-demethoxygeldanamycin (alvespimycin, 17-DMAG) are geldanamycin-derivatives; 17-DMAG is a water-soluble drug, and 17-AAG has an improved formulation (DMSO-free). Retaspimycin and resorcinol derivatives, as many other HSP inhibitors, are still under clinical investigation for the treatment of colorectal, breast, and other cancers [116]. Growing evidence indicates that HSP90 inhibitors could be beneficial in the treatment of both myeloproliferative neoplasms and inflammatory reactions. However, most of the developed HSP90 inhibitors have hepatotoxicity, nephrotoxicity, and gastrointestinal toxicity [116].

In inflammatory reactions, HSP90 inhibition leads to the inhibition of the NLRP3 inflammasome release, because inactive intracellular protein NLRP3 is folded by HSP90. Upon stress and induction of HSP90, NLRP3 is activated and promotes inflammation. It was shown that, after HSP90 blocking with geldanamycin treatment, the activation of the inflammasome was blocked. NLRP3 released from its protective complex became degraded by autophagy or secreted from the cells. Thus, the controlled destruction of NLRP3 is a potential pathway to regulate the chronic inflammation, particularly in the development of age-related macular degeneration (AMD), as recently shown [118]. The inhibition of the NLRP3 inflammasome pathway by P_4_ has also been shown [81].

P_4_ and progestins have not been intensively explored as a potent modulator or inhibitor of HSP90 expression. Nevertheless, it is assumed that P_4_ and progestins can specifically regulate HSP90 activity. This is supported by a number of studies, which have convincingly demonstrated that P_4_ blocks estradiol-stimulated HSP90 expression. Therefore, P_4_ did not affect the amount of Hsp90 and Hsp70 mRNA, but inhibited their increase stimulated by estradiol, as was shown in uterine tissues of ewes [119].

A similar inhibitory effect of P_4_ on Hsp90 mRNA expression was observed in normal samples of human endometrial and myometrial tissues over the whole menstrual cycle and in short-term cultures of steroid-responsive (T47-D) and unresponsive (HRT-18) cell lines exposed to estradiol and P_4_ over a 24 h incubation period [120]. In these cases, P_4_ alone had no significant effect on the expression of Hsp90 and Hsp70 mRNA but inhibited the estradiol-stimulated increase in their expression. As a result of this suppression, Hsp90 mRNA in the endometrium and Hsp70 mRNA in the myometrium and endometrium were no longer significantly elevated above control levels. Interestingly, no clear effect of agonists on Hsp mRNA expression was apparent in HRT-18 cultures, which are insensitive to P_4_ and estradiol [120].

On the basis of these results, it can be proposed that P_4_ predominantly inhibits the estradiol-stimulated HSP90 expression in hormone-sensitive tissues.

A more recent study on ovariectomized macaques showed an increase in the level of HSP90 after progesterone and estradiol treatment. Presumably, this action is nonspecific, since neuronal cells are not straight targets for ovarian steroids [121].

Since HSP90 clients are involved in multiple signaling pathways ranging from TGF-β and mitogen-activated kinase (MAPK) to TNF-α, G-protein-coupled receptor (GPCR), and calcium signaling, the inhibition of Hsp90 suppresses their activation. Consequently, these pathways can be inhibited by P_4_. If further studies can confirm the inhibition of HSP90 by P_4_, this approach could also be very beneficial in the treatment of heart diseases [122].

## 6. Other Evidence of the Anti-Inflammatory and Immunomodulatory Action of Progesterone

### 6.1. The Influence on Chemokine Production 

A number of studies have shown that P_4_ and synthetic progestogens are able to suppress the production of chemokines and change chemokine–chemokine receptor profiles. P_4_ (10 µM) caused a 50–70% inhibition of RANTES, MIP1α, and MIP1β secretion in CD8^+^ cells of the peripheral blood mononuclear fraction [123].

Specific downregulation of the monocyte chemoattractant protein-1 (MCP-1) has been observed in human endometrial stromal fibroblast cells under the action of P_4_ and MPA, LNG, and NETA, progestins structurally related to progesterone and testosterone. All progestins downregulated not only MCP-1, but also IL-6, TGFβ1, and matrix metalloproteinase-3 in this culture [124].

The role of contraceptive intravaginal progestins in possible protection against HIV is currently being actively investigated. It is assumed that P_4_ protects against HIV-1 infection by reducing the expression of CCR5, the main co-receptor for HIV entry into human cells. The synthetic progestins etonogestrel and MPA increase the rate of virus penetration, although this may be due to the thinning of the endometrium [125].

### 6.2. Inhibition of WNT and MAPK Pathways

Recently it has been shown that the main role in inflammation in endometriosis is played by MAPK and WNT/β-catenin cascades. Since endometriosis is a chronic inflammatory disease, their inhibition may be a therapeutic strategy for endometriosis [126]. Progestins at high dosage inhibit both MAPK and WNT/β-catenin pathways; therefore, this effect may also be one of the mechanisms of their anti-inflammatory action [127,128].

### 6.3. Diminution of Estrogen Action by P_4_

E2 has proinflammatory and mitogenic properties [93]. COX-2 promotes mammary adipose tissue inflammation, local estrogen biosynthesis, and carcinogenesis by inducing the secretion of cytokines and prostaglandins from peritoneal macrophages [129]. Estrogen receptors, particularly ER-β, are known to interact with some inflammasome components such as NLRP3 sensor and caspase 1 to activate IL-1β production. P_4_ downregulates ER, representing an additional anti-inflammation mechanism [130].

### 6.4. GR-Mediated Anti-Inflammatory Action of Progestins 

The anti-inflammatory effects of progestins may be associated with partial agonism of GR if they have agonistic activity toward GR, as in the case of MPA. Thus, in the endocervical epithelium cell line model, MPA, unlike NET-acetate and P_4_, increased mRNA expression of the anti-inflammatory GILZ and IκBα genes [131]. Similarly, MPA, unlike NET-A, decreased the expression of the proinflammatory IL-6, IL-8, and RANTES genes, as well as the level of IL-6 and IL-8 proteins. Marinello et al. also demonstrated an anti-inflammatory GR-mediated action of MPA [75]. In primary human amnion mesenchymal cells, MPA had an anti-inflammatory effect via the inhibition of IL-1β-induced MMP-1 and IL-8 expression. This effect of MPA was blocked with the inhibition of glucocorticoid receptor expression by small interfering RNA transfection [75].

## 7. New Aspects in the Application of Progesterone and Progesterone Receptor Chaperones to Reduce Inflammation

The anti-inflammatory action of P_4_ was described above, and the main mechanisms were shown on Figure 1. An additional intriguing aspect is the possibility to potentiate the P_4_ anti-inflammatory action by targeting PR chaperones, such as immunophilins. As immunophilins are involved in the formation of active progesterone receptor complexes, they can regulate the specific pharmacological activity of progesterone, androgens, and glucocorticoids [100].

Immunophilins FKBP52 and FKBP51 are Hsp90-associated cochaperones. However, if HSP90 inhibitors have a broader spectrum of action, since HSP90 folds a great number of signal proteins in the cell (so-called “clients”), then FKBP52 or FKBP51 inhibitors should be much more specific and their influence, should not be global across the organism.

The significance of FKBP52 and FKBP51 was recently demonstrated in animal models with the knockout of these genes. Mice deficient in the FKBP52 gene were insensitive to androgens, glucocorticoids, and progesterone, but no more severe effects were observed. Thus, after inhibiting FKBP52, the action of these steroid hormones could be carefully eliminated when necessary. Moreover, a pharmacological target in FKBP52 has already been established, i.e., a proline-rich loop in the FK1 catalytic domain [132,133,134]. As for FKBP51/FKBP52 double-knockout mice, embryonic lethality took place in all cases [132].

FKBP52 is a validated target of the clinically approved immunosuppressive drug, FK506 (tacrolimus). Thus, the development of FKBP52-specific small-molecule inhibitors is predicted to be a highly targeted strategy with potential for the treatment of any disease that is dependent on functional AR, GR, and/or PR signaling pathways. Thus, the action of progestins, androgens, and GK can be enhanced by tacrolimus. In this way, it is possible to decrease the dosage of the appropriate hormone and the FK506 immunosuppressant. 

With regard to HSP90 inhibitors, their elaboration is also a fast-developing pharmaceutical direction, with several drugs in clinical trials, most of which are aimed at anticancer therapy [134,135]. However, in some cases, small-molecule inhibitors of Hsp90 have been found to be cardiotoxic. Considering this connection, specific targeting of Hsp90 via modulation of post-translational modifications (PTMs) seems to be a more appropriate strategy [88]. Surprisingly, increased HSP expression and functioning may even be protective for the myocardium. The therapeutic enhancement of intracellular HSP activity with commercially available drugs (e.g., geranylgeranyl acetone, an antiulcer drug that supports HSP70) has been experimentally shown to provide protection against myocardial infarction [136].

For FKBP51 immunophilin targeting, there are not as many intensively explored mechanisms. As described above, FKBP51 expression has a dual role in inflammation and stress-related signaling; specifically, it is positive in protecting against oxidative stress and negative in the case of already activated NF-κB-related gene networks [137]. FKBP51 overexpression protects cells against oxidative stress, while FKBP51 knockdown makes them more sensitive to injury [109]. 

It should be noted that there are strong interactions between HSP90 and FKBP51, since Hsp90 inhibitors promote the reversible translocation of FKBP51 from the mitochondria to the nucleus. These data suggest that the unexpected mitochondrial localization of FKBP51 may be related to its antiapoptotic effect [109].

P_4_ also demonstrated a unique action on mitochondria [91]; thus, considering the expression of FKBP51, together with the finding that mitochondrial PR also exists in mitochondria [138], the translocation of FKBP51 from the mitochondria to the nucleus may have great importance. Hsp90 inhibitors favor FKBP51 translocation from the mitochondria to the nucleus in a reversible manner. After radicicol treatment, FKBP51 no longer resides in the mitochondria and concentrates in the nucleus. When the situation returns to normal, FKBP51 rapidly cycles back to the mitochondria, completing its cycle. Because both p53 and NF-κB are mitochondrial proteins, it may be that FKBP51 interacts with them in the mitochondria [109].

In inflammatory diseases, both the activation of the NF-κB pathway and the activation of the p53 pathway can occur [139]. P53 is required for the suppression of NF-κB by anti-inflammatory drugs, particularly GC and possibly P_4_. Loss of p53, a tumor suppressor protein, impairs transcriptional repression of the NF-κB target gene by glucocorticoids. Thus, p53 plays a key role in the repression of NF-κB [140].

P53 and the GR are transported to the nucleus in a similar manner. The complexes of steroid hormone receptors with HSP90 and FKBP52 are transported to the nucleus by dynein, while the p53 complex with HSP90 and FKBP52 is transported in the same way upon DNA damage and cellular stress [141,142].

The identity transport of steroid receptor complexes and P53 complexes with immunophilins into the nucleus indicates that pharmacological regulation of this transport can reduce or induce the transcription of the corresponding genes.

The trafficking from the nucleus to the mitochondria is an important process, and it can be pharmacologically regulated. It was noted that toxicants induced an accumulation of NF-κB and p53 proteins in the nucleus, while the inhibitor of NF-κB nuclear translocation SN50 blocked the increase of p53 expression and suppressed the expression of *COX-2* and TNF-α [139]. The further exploration of the role of mitochondrial PR in oxidative stress, injury, and inflammation can open up new perspectives for regulation by targeting PR chaperones. This can be especially useful in the treatment of neurological and cardiac diseases.

More and more publications are appearing with respect to FKBP51 as a stress-related protein [143]. When the stress-related protein FKBP51 partners with Hsp90, this formidable chaperone protein complex prevents clearance from the brain of the toxic tau protein associated with Alzheimer’s disease. Hsp90 supervises the activity of tau inside nerve cells. Chaperone proteins typically help ensure that tau proteins are properly folded to maintain the healthy structure of nerve cells [139].

Polymorphisms in FKBP51 have been associated with a number of neurological conditions including depression, suicide, and Alzheimer’s disease [144]. Therefore, FKBP51 is a new treatment target for diseases with tau pathology. FKBP51 emerged as a key player in several diseases such as stress-related disorders, chronic pain, and obesity. The historically first FKBP51 inhibitor FK506 was shown to have neuroprotective effects and induce neuronal regeneration, suggesting significant roles for FKBPs in neural growth, injury, and regeneration. Linear analogues of FK506 called SAFit were shown to be highly selective for FKBP51 over its closest homolog FKBP52, allowing proof-of-concept studies in animal models.

Recently, newly synthesized macrocyclic inhibitors in complex with FKBP51 showed the desired selectivity-enabling binding mode, confirming that macrocyclization is a viable strategy to selectively target the shallow FKBP51 binding site [145].

As FKBP51 associates with HSP90 and appears in functionally mature steroid receptor complexes [146], the universal molecules for their inhibition are P_4_, progestins, or other known PR ligands. In this case, tacrolimus and cyclosporine may potentiate the action of progestins.

Tacrolimus also affects various physiological functions, especially those mediated by glucocorticoid receptors [102]. Improvement in systemic and ovarian immune function, expression of endometrial progesterone receptors and co-receptors, and adaptation of uterine vessels to pregnancy were signs of increased sensitivity of progesterone receptors in a mouse model of the disease treated with low doses of tacrolimus. It is possible that these effects are mediated by the restoration of P_4_ insensitivity [101]. A clinical trial of cyclosporine in RSA was recently initiated in China. As a background for its implementation, the authors suggest that CsA in the cytoplasm forms a special complex with cyclophilin that binds calcineurin phosphatase and prevents lymphocyte proliferation, as well as the transcription of lymphocyte factors (TNF-α, IL-2, and IFN-γ), by inhibiting serine threonine protein phosphatase activity, which leads to immunosuppression. Thus, CsA may improve pregnancy outcomes in women with RSA, primarily in those with elevated Th1 immune-response phenotypes [147]. Combinations of progestins with CsA or tacrolimus require further preclinical studies devoted to the P_4_ resistance.

Another little-explored role of in the suppression of inflammation is related to the fact that the specific target of progesterone action is the kappa-type opioid receptor agonist [148], the activation of which reduces pain, one of the main signs of inflammation. Through the action of its agonist on this receptor, P_4_ may help reduce chronic pain, as observed in endometriosis, rheumatoid arthritis, and other inflammatory diseases. Although this assumption is based on a small number of publications [149,150], the study of the regulation of the kappa-type opioid receptor by P_4_ appears to be important in understanding the role of P_4_ in the regulation of inflammation.

## 8. Conclusions

The analysis of the current data revealed the key fundamental mechanisms through which P_4_ can be used in the treatment of hormone-resistant chronic inflammatory diseases.

Specific mechanisms of P_4_ anti-inflammatory and immunomodulatory action are associated with the regulation of T lymphocytes and the inhibition of proinflammatory cytokines and chemokines, as well as the phenomenon of immune tolerance and the involvement of coactivators and cochaperones of progesterone, androgen, and glucocorticoid receptors. Moreover, the specific anti-inflammatory influence of P_4_ and progestins includes the antagonistic action against estrogen receptor beta-mediated signaling and inhibition of WNT, MAP, and TGFβ signaling pathways.

Steroid receptor cochaperone HSP90 and immunophilins FKBP51 and FKBP52 play an important role in the intracellular signaling of steroid hormones, including progesterone. They have an opposite action. FKBP52 is a positive regulator of the glucocorticoid receptor, progesterone receptor, and androgen receptor, but not the estrogen receptor or mineralocorticoid receptor. FKBP51 is a negative regulator of steroid hormone activity, as reported in most studies. By regulating the activity and expression of FKBP51 and FKBP52, it is possible to increase or decrease hormonal signaling, as well as restore it during the development of hormone resistance [151].

HSP90 inhibitors can restore the lost anti-inflammatory effect of GC and P_4_ in the event of resistance, which is important in chronic inflammatory diseases such as endometriosis, psoriasis, atopic dermatitis, and rheumatoid arthritis. The ligands of the immunophilins FKBP51 and FKBP52—tacrolimus and cyclosporine—can act in a similar way. The immunomodulatory and anti-inflammatory effects of tacrolimus, cyclosporine, and HSP90 inhibitors are characterized, among other factors, by their participation in the formation of an active ligand–receptor complex of progesterone and interactions with its components—immunophilins FKBP51 and FKBP52. Progesterone itself activates the production of the progesterone-induced blocking factor (PIBF) and LIF, which have multiple anti-inflammatory effects, inhibiting the activation of T lymphocytes and decreasing the production of proinflammatory cytokines.

P_4_ has systemic immunomodulatory effect that is realized through the membrane [68,152] and classical nuclear receptors [87,153,154,155,156] in immune cells. Immune cells express both membrane and nuclear PRs [157,158], and their expression profile depends on the type of immune cells, microenvironment, and other factors, which should be clarified in further studies. The systemic immunomodulatory effect of P_4_ can be also explained by the presence of the PRs in the venous endothelium, where PR signaling directly regulates cytokine expression [159].

Combinations of FDA-approved tacrolimus or cyclosporine with progesterone and its analogues can effectively suppress FKBP51, their common target. Tacrolimus firstly binds to FK506-binding protein or FKBP12, which regulates specific cell signaling pathways such as mTORC1, calcineurin-activated NFAT, and TGFβ receptor signaling. After FKBP12 binds with tacrolimus or CsA, this complex interacts with FKBP51 or FKBP52, which regulate each other’s expression in a feedback manner. Both regulate the active state of the hormone–receptor complex. The pharmacological regulation of progesterone receptor cochaperones may be a promising strategy for the treatment of progesterone-resistant chronic inflammation. Thus, pharmacological regulation with P_4_ as an anti-inflammatory drug can be used in chronic inflammation processes with concomitant GC and P_4_ resistance, as well as in the treatment of COVID-19 patients with low concentrations of P_4_ (menopausal women and older men). GC or P_4_ treatment can be combined with tacrolimus, which increases the sensitivity to these hormones.

## Figures and Tables

**Table 1:** The examples of anti-inflammatory action of progesterone and progesterone- related drugs on different human and animal models.

P_4_ or Progestin	Concentration/Dosage	Inflammatory Process	Spices	Ref.
P_4_	2 mg/kg	sepsis syndrome	rat	[24]
P_4_	150 mg/kg	retinitis pigmentosa (RP)	mice	[25]
P_4_	100 mg/kg	LPS-induced pregnancy loss	mice	[26]
P_4_	5 ng/mL	LPS- or Escherichia coli-stimulated bovine endometrial stromal cells	cow	[27]
P_4_	16 mg/kg	rat temporomandibular joint disorder inflammation	rat	[28]
Nestorone	10 μg/kg–80 μg/kg	multiple sclerosis (MS), amyotrophic lateral sclerosis (ALS), spinal cord injury (SCI)	human	[29]
MPA	10 μm	human PBMC, stimulated with PHA and the antigen Streptokinase	human	[30]
Dienogest	1 μm	inflammatory reaction of human endometrial epithelial cells in vitro	human	[31]
MA	160 mg/d	human anorexia, cachexia or unexplained weight loss	human	[32]
Tibolon	0.01 μM	BV-2 microglia cells treated with palmitic acid	mice	[33]

**Table 2:** Progesterone-regulated proinflammatory and anti-inflammatory cytokines.

Proinflammatory	Main Functions in Inflammation	Ref.
IL-1β	Activation of lymphocytes and macrophages, stimulation of T-cell proliferation, increase in the adhesive activity of leukocytes, production of acute phase proteins, fever induction	[70,71,72]
IL-2	Activation of T cells, start and maintenance of their proliferation, stimulation of proliferation of NK and B cells	[73]
IL-6	Differentiation of the B lymphocytes, induction of acute phase proteins	[74]
IL-8	Induction of chemotaxis, release of oxygen radicals and degranulation, angiogenesis induction	[75]
TNFalpha	Activation of macrophages, granulocytes, cytotoxic T-lymphocytes, increase in the adhesion of leukocytes to the endothelium, increase in fever and cachexia, induction of acute phase proteins, angiogenesis, increase of the expression of MHC 1	[76]
IFNγ	Increase in the expression of MHC 1 and 2 molecules, activation of macrophages, increase in the adhesion of leukocytes to the endothelium, antiviral and antiproliferative effects	[54]
Anti-inflammatory	Main functions in inflammation	
IL-1ra	Specific inhibition of IL-1a- and IL-1b-mediated cellular activation at the IL-1 cellular receptor level	[77]
IL-4	Increased B lymphocyte proliferation, B lymphocyte growth factor, antagonism with IFNγ, promotion of Th2 lymphocyte development; inhibition of LPS-induced proinflammatory cytokine synthesis	[78]
IL-10	Inhibition of the proinflammatory cytokine synthesis, inhibition of Th1-type lymphocyte responses	[74]
TGFβ	Suppression of the production of pro-inflammatory cytokines, stimulation of the repair, fibrosis	[35]

## Data Availability

Not applicable.

## Abbreviations

AR, androgen receptor; CTL: cytotoxic T lymphocyte(s); El, estrone; E2, 17β-estradiol; EMT, epithelial–mesenchymal transition; ER, estrogen receptor; GR, glucocorticoid receptor; GC, glucocorticoids; HCC, hepatocellular carcinoma; IL, interleukin; MCP-1/CCL2, monocyte chemoattractant protein-1/2; MPA, medroxyprogesterone acetate; MPN, myeloproliferative neoplasms; mPR—membrane PR; MR, mineralocorticoid receptor; NETA, Norethisterone acetate; NK, natural killer; NLRP3, NOD-, LRR-, and pyrin domain-containing protein 3; NHE1, Na(+)/H(+)-exchange 1; NFAT1, Nuclear Factor of Activated T-Cells 1; NFKB, nuclear factor kappaB; P_4_, progesterone; PIBF, progesterone-induced blocking factor; PHA, phytohemagglutinin; PBMC, peripheral blood mononuclear cell; PGE2, prostaglandin E2; PR—progesterone receptor; RT, respiratory tract; SOCS, suppressors of Cytokine Signaling(SOCS) proteins; TNFα, tumor necrosis factor alpha; TGFβ, Transforming growth factor beta; Tregs, regulatory T cells.
